# Compact and cost-effective laser-powered speckle contrast optical spectroscopy fiber-free device for measuring cerebral blood flow

**DOI:** 10.1117/1.JBO.29.6.067001

**Published:** 2024-05-31

**Authors:** Yu Xi Huang, Simon Mahler, Maya Dickson, Aidin Abedi, Julian Michael Tyszka, Yu Tung Lo, Jonathan Russin, Charles Liu, Changhuei Yang

**Affiliations:** a California Institute of Technology, Department of Electrical Engineering, Pasadena, California, United States; b University of Southern California, USC Neurorestoration Center, Department of Neurological Surgery, Los Angeles, California, United States; c California Institute of Technology, Division of Humanities and Social Sciences, Pasadena, California, United States; d Rancho Los Amigos National Rehabilitation Center, Downey, California, United States

**Keywords:** speckle contrast optical spectroscopy, cerebral blood flow, laser speckle imaging, biomedical optics, noninvasive brain imaging, diffuse correlation spectroscopy

## Abstract

**Significance:**

In the realm of cerebrovascular monitoring, primary metrics typically include blood pressure, which influences cerebral blood flow (CBF) and is contingent upon vessel radius. Measuring CBF noninvasively poses a persistent challenge, primarily attributed to the difficulty of accessing and obtaining signal from the brain.

**Aim:**

Our study aims to introduce a compact speckle contrast optical spectroscopy device for noninvasive CBF measurements at long source-to-detector distances, offering cost-effectiveness, and scalability while tracking blood flow (BF) with remarkable sensitivity and temporal resolution.

**Approach:**

The wearable sensor module consists solely of a laser diode and a board camera. It can be easily placed on a subject’s head to measure BF at a sampling rate of 80 Hz.

**Results:**

Compared to the single-fiber-based version, the proposed device achieved a signal gain of about 70 times, showed superior stability, reproducibility, and signal-to-noise ratio for measuring BF at long source-to-detector distances. The device can be distributed in multiple configurations around the head.

**Conclusions:**

Given its cost-effectiveness, scalability, and simplicity, this laser-centric tool offers significant potential in advancing noninvasive cerebral monitoring technologies.

## 1 Introduction

Monitoring the cerebral blood flow (CBF) has broad significance in both clinical settings and cognitive neuroscience research.^1^^,^^2^ Measuring CBF noninvasively poses a persistent challenge, primarily attributed to the difficulty of accessing and obtaining signal from the brain, especially in biomedical context where exposure levels are restricted for the safety of the subject.^3^ As a result, efforts have been devoted in diverse methods for measuring CBF. Some notable techniques include transcranial Doppler ultrasound,^4^^,^^5^ magnetic resonance imaging (MRI),^6^^,^^7^ near-infrared spectroscopy,^8^^,^^9^ electroencephalography,^10^ and cerebral oximetry. Optical monitoring of CBF has potentially higher sensitivity and temporal resolution than other techniques^11^ and is generally more cost effective than ultrasound and MRI.

Diffusing wave spectroscopy utilizing laser light transmitted through a scattering medium to extract the dynamic information has recently garnered attention as a promising tool for CBF monitoring.^12^^–^^18^ One advantage of diffusing wave spectroscopy is the capability to collect a substantial number of photons that have interacted with the brain. It also presents numerous operational benefits including its nonionizing radiation, straightforward methodology, use of relatively lightweight and cost-effective equipment, and compatibility with advanced commercial optical systems that can be readily adapted. In diffusing wave spectroscopy scheme, laser light is injected into the head using a laser source, and the emerging light is collected by a detector positioned at a source-to-detector (S–D) separation distance from the injection spot. The movements of blood cells within the travelling light’s path will scatter and change the effective optical path lengths, resulting in a fluctuating laser speckle field.

There exist two types of sampling techniques to infer the blood flow (BF): temporal and spatial. The temporal sampling technique, time-domain diffuse correlation spectroscopy, is based on the use of the temporal ensemble of the speckle field and uses a detector working at a high frame rate (typically above 100 kHz) on a single (or on a small group of) speckle(s).^12^^,^^13^ The spatial sampling technique is an off-shoot of laser speckle contrast imaging (LSCI) and is based on the use of spatial ensemble of the speckle field. In the spatial ensemble, instead of using a detector at a high frame rate, a camera with a larger detecting area and a large number of pixels is used to collect more photons and speckles within the same frame.^16^^,^^17^^,^^19^ The camera is typically working at an exposure time longer than the decorrelation time of the speckle field. This results in multiple different speckle patterns summing up onto a single camera frame. As the speckle field fluctuates, the speckle pattern recorded by the camera is smeared and washed out within the exposure time. Because the smearing or the washing out effect is primarily due to the dynamics of the blood cells, the decorrelation time can be calculated from the degree of blurring of the captured frame, typically by calculating the speckle contrast. Based on the spatial sampling technique, compact and wearable systems for measuring BF noninvasively and continuously via speckle contrast calculations have been developed.^16^^,^^18^^,^^20^^–^^27^ There exist two main techniques for the speckle contrast calculations. The first one is to use a sliding window for speckle contrast calculations, typically of size 7×7  pixels, in the camera images.^21^^–^^26^ This technique proved effective for BF measurement when the detected signal level is above the camera noise level. The second technique is to use the entire camera image, typically with more than a million pixels, for speckle contrast calculations, which proved effective for BF measurement when the detected signal is close or even below the camera noise level, such as for measuring cerebral BF at long S–D >3  cm distances.

This technique is usually referred as speckle visibility spectroscopy (SVS),^16^^,^^17^^,^^19^^,^^28^^,^^29^ or as diffuse speckle contrast analysis (DSCA),^30^^,^^31^ or as speckle contrast optical spectroscopy (SCOS).^20^^,^^32^^–^^34^ The terminology SVS was first introduced by Bandyopadhyay et al.,^29^ DSCA by Bi et al.,^31^ and SCOS was introduced by Valdes et al.^34^ However, SCOS terminology is more commonly used than SVS and DSCA for describing devices measuring cerebral BF via speckle contrast calculations. For clarity, we will use the term SCOS throughout. In order to measure CBF noninvasively, we need to adjust the system, so that the system detects a larger portion of signal from the brain than the scalp + skull layers. The brain sensitivity significantly increases with the depth of penetration, attained by increasing the S–D but at the cost of a lower signal-to-noise ratio (SNR).^13^^,^^14^^,^^17^^,^^35^^,^^36^ At S–D >3  cm, the brain sensitivity was reported to be larger than the scalp + skull layers. SCOS was applied on the human head to monitor CBF noninvasively, allowing for the detection of a larger number of speckles and an increased proportion of detected light from the brain.^16^^–^^18^^,^^20^

This paper reports a compact SCOS device designed for monitoring relative cerebral BF at long S–D distances (>3  cm). This wearable sensor module hardware consists solely of a continuous-wave laser diode and a high-resolution CMOS-based board camera that can be easily placed on a subject’s head to measure BF with no external optical elements. It offers real-time BF monitoring with a sampling rate of 80 Hz while maintaining a lightweight and budget-friendly design. While a similar wearable optical system was recently used to measure the changes of BF during breath hold maneuver,^20^ this paper presents the design and processing of compact SCOS and compares its gain in stability and SNR over single-fiber-based SCOS. The compact SCOS device is composed of a laser diode (source unit) controlled by a lightweight and compact laser diode driver (powered by a 9-V battery), which can be encased in a wearable sensor module. The detecting unit is composed of a CMOS camera connected to a computer via USB. Although the wired connection to the computer restricts full portability, we envision the camera connectivity to the computer becoming wireless, enabling the detector unit to be both compact and lightweight for wearable use. The total cost of the compact SCOS device is approximately $500 (laptop not included).

Here we demonstrate that compact SCOS has certain advantages over the single-fiber-based one. First, it achieves better SNR compared with single-fiber-based SCOS, collecting a larger number of photons due to a significant increase in the detecting area and numerical aperture (NA). Specifically, we measured the compact SCOS version to capture about 70 times more signal relative to a 600-μm single-fiber-based SCOS device, improving detectability of CBF at extended S–D >3  cm distances. Second, it eliminates the motional artifacts associated with the light guide running from the head to the camera, showing a superior stability and reproducibility. Typically, when a large-diameter multimode fiber is used to collect the photons from the head to the camera, slight movements of the fiber can cause significant speckle changes, disrupting the results.^17^^,^^18^^,^^28^ This issue is currently mitigated by minimizing fiber perturbations with extraordinary measures, which is not ideal.

The paper is organized as follows. First, we detail the design and experimental arrangement of the proposed compact SCOS system and describe the data processing for calculating BF from the recorded camera images. Second, we compare the performance of compact SCOS and single-fiber SCOS using static and moving phantoms. Finally, we experimentally compare the BF measured from compact SCOS and single-fiber SCOS devices at different S–D distances from a cohort of five human subjects. Our results show significant improvements in BF measurement with the compact SCOS over the single-fiber SCOS device.

## 2 Methods

**Fig. 1:**
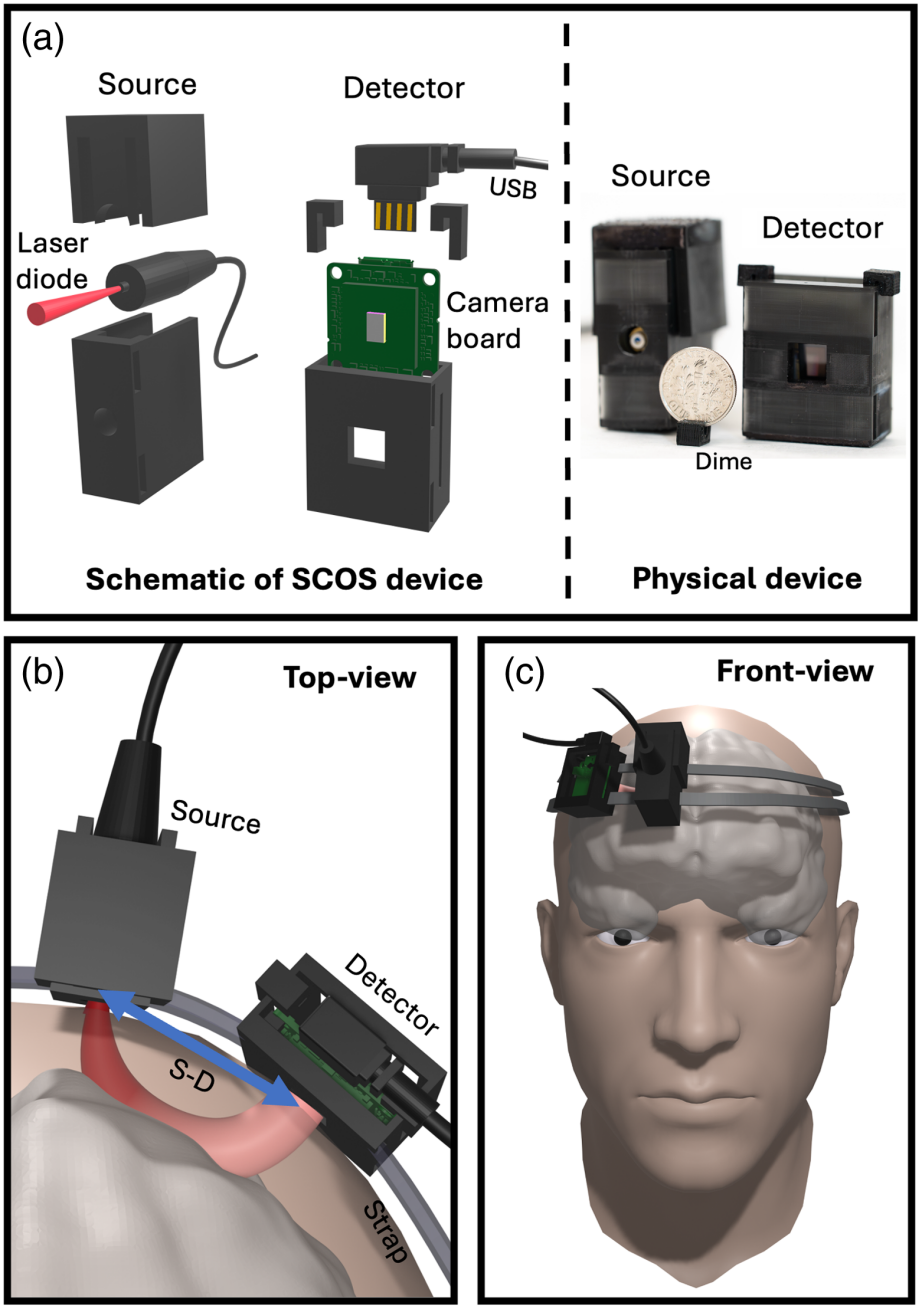
Compact SCOS setup. (a) Design of the SCOS device, consisting of a laser diode (source) and a CMOS-based board camera (detector) both housed in a 3D-printed mount. (Left panel) 3D schematics breakdown and (right panel) photograph of the actual SCOS device. (b) Top-view and (c) front-view schematics illustrating the SCOS device in use on a subject’s forehead. When set at a specific S–D distance, the SCOS device can effectively measure CBF.

The arrangement of our compact SCOS device is shown in Fig. 1. The system design is shown in Fig. 1(a) with the schematics shown on the left and a photograph of the 3D-printed device shown on the right. The system includes a laser source for illumination and a board camera for detection. A dime is included in the photograph for size comparison.

For this study, we used a single-mode continuous wave 785-nm laser diode as a source [Thorlabs L785H1], which can deliver up to 200 mW. To ensure control over the illumination spot size and prevent undesirable laser light reflections or stray light, we housed the laser diode within a 3D-printed mount with a circular aperture of 5 mm. We also housed the camera in a 3D-printed mount. The mounts were printed using black resin which absorbs light, minimizing back reflection and stray light. The laser diode was set several millimeters away from the skin of participants such that the illumination spot diameter was 5 mm.^17^ The total illumination power was limited to 45 mW to ensure that the laser light intensity level of the area of illumination is well within the American National Standards Institute laser safety standards for maximum permissible exposure ($2.95\;\mathrm{mW}/{\mathrm{mm}}^{2}$) for skin exposure to a 785-nm laser beam^3^ (see Supplementary Material for more details about the study participation).

At a S–D distance from the illumination spot, the detector was positioned on the head of the subject to collect the emerging light away from the laser illumination spot [Figs. 1(b) and 1(c)]. The collected laser light was directed onto a carefully selected camera equipped with a large sensor area and small pixel size, maximizing the number of speckles captured. We used a USB-board camera [Basler daA1920-160um (Sony IMX392 sensor)]. For optimal performance and stability, we typically operated the camera at a framerate of 80 frames-per-second (fps). The compact SCOS system has the potential to achieve a sampling rate of up to 160 fps. However, it is capped at 80 fps to provide a balance between storage space and temporal resolution. To ensure time-synchronization among all pixels, the camera was configured with a global shutter setting. The camera features a pixel pitch of 3.4  μm, which offers a balance between the average intensity per pixel and the number of speckles per pixel, which was estimated to be about 10 speckles per pixels, corresponding to a one-dimensional speckle-to-pixel length ratio of s/p=0.3. Although an s/p ratio of two or above is typically used to perform speckle imaging, here we are interested in extracting the dynamics of the scattering medium via speckle statistics. Toward this end, it has previously been shown that maximum SNR can occur at s/p ratio close or lower than one^16^^,^^37^^,^^38^ (see Supplementary Material for more details).

**Fig. 2:**
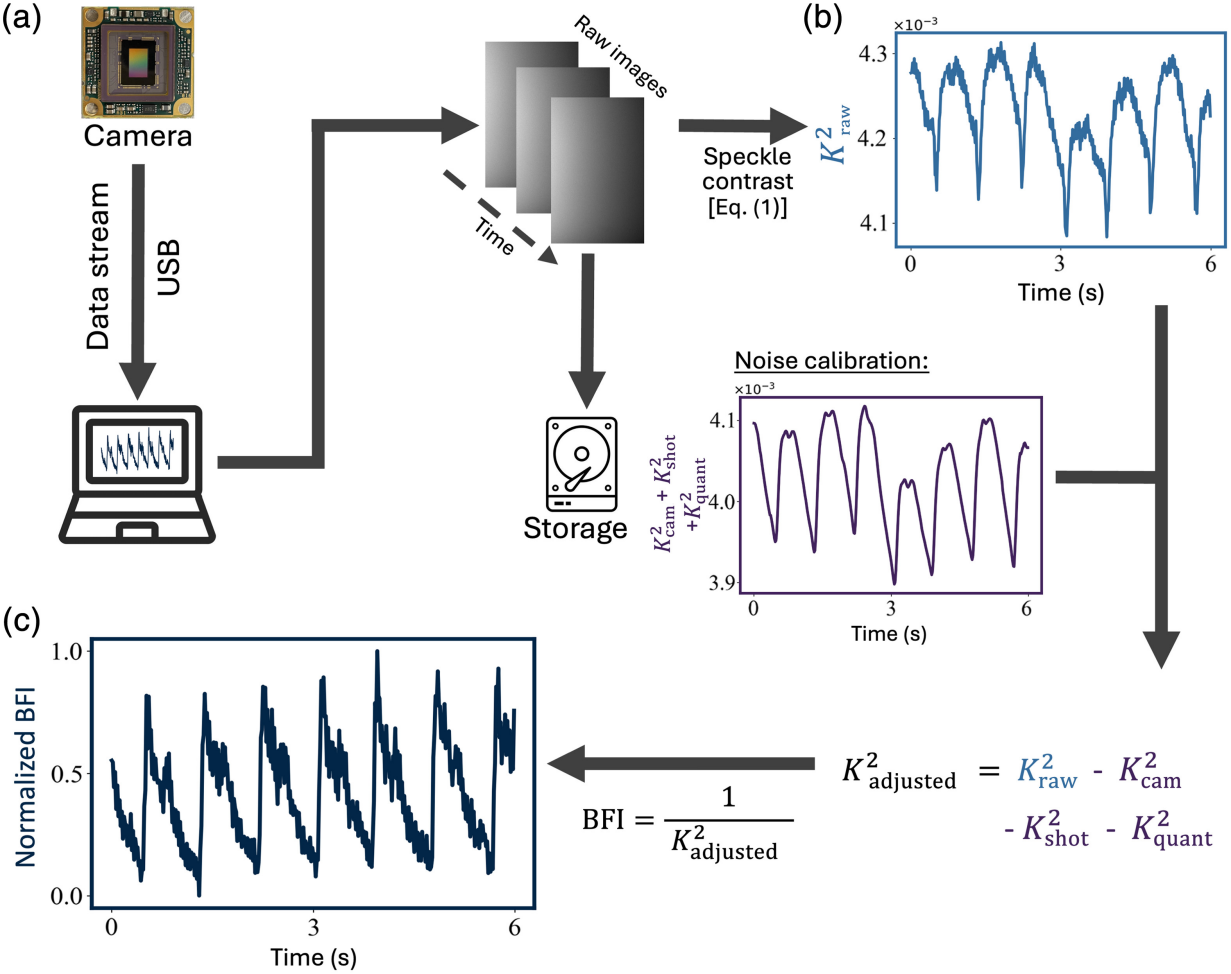
Compact SCOS processing analysis flowchart for deriving the blood flow from recorded camera images. (a) Recording and storing of SCOS camera images. (b) Measured raw speckle contrast calculated from the images in (a). (c) Calculated BFI after calibrating the raw speckle contrast in (b).

The depth to which the photons have traveled deep into the head is related to the S–D distance.^14^^,^^17^ By tuning the S–D distance, one can tune the depth of penetration into the head, where a banana-shaped spatial sensitivity of the light path is usually observed as shown in Fig. 1(b).^14^^,^^17^ As the S–D distance increases, the banana shape extends deeper into the brain, with deeper brain regions being more challenging to access. The spatial distribution of the exiting photons collected by a camera exhibits a granular pattern with areas of high and low intensity called speckles. Speckles arise from interference between the numerous random scatterings with the coherent light field and constitute a vast area of research.^39^ We image these speckles onto a camera with a finite exposure time [Fig. 2(a)]. The camera must operate at a high enough frame rate to temporally resolve the dynamics, typically above 20 fps for BF measurements. Speckles undergo dynamic changes with a specific temporal evolution,^40^^–^^42^ characterized by the decorrelation time $\tau_{c}$ of the speckle field.^43^^,^^44^ The camera is configured with an exposure time T that is significantly larger than the decorrelation time $\tau_{c}$. The motions within the light paths, primarily due to the movement of red blood cells, will scatter and change the effective optical pathlengths resulting in a fluctuating speckle field that varies in time. As the speckle field fluctuates, the recorded speckled image would be smeared and washed out: the shorter the speckle decorrelation time, the more washed out the image. The dynamics of the speckles can be quantified by calculating the speckle contrast of the recorded image.

The experimental SCOS processing analysis flowchart for deriving the BF from recorded camera images is shown in Fig. 2. The first step is to remove the nonuniform intensity distribution and from the camera images (normalization). Since one side of the camera sensor is closer to the laser source, the recorded intensity at pixels closer to the source will have a higher readout than those further away (see Supplementary Material for more details about the normalization method). Then the squared speckle contrast $K_{\text{raw}}^{2}(I)$ of the normalized image [Fig. 2(a)] is calculated as

$$K_{\text{raw}}^{2}(I)=\frac{\sigma^{2}(I)}{\mu^{2}(I)}.$$ (1)

The variance of the normalized image I is $\sigma^{2}(I)$ and its mean is μ(I). This calculation does not account for noises that contribute to the variance of the images. To account for these noises, we use an adjusted squared speckle contrast $K_{\text{adjusted}}^{2}$, which is commonly calculated as^18^^,^^45^^–^^47^

$$K_{\text{adjusted}}^{2}=K_{\text{raw}}^{2}-K_{\text{shot}}^{2}-K_{\text{quant}}^{2}-K_{\mathrm{cam}}^{2},$$ (2)

with $K_{\text{shot}}^{2}$ accounting for variance contributions from the shot noise, $K_{\text{quant}}^{2}$ for the variance inherited from quantization, and $K_{\mathrm{cam}}^{2}$ for the variance contributions of the camera’s readout noise and dark noise [see Fig. 2(b) for examples of raw and noise speckle contrast measurements]. For each of the image I, they can be calculated as the following:^18^^,^^45^^–^^47^

$$K_{\text{shot}}^{2}(I)=(\frac{\gamma }{\mu (I)}),$$ (3a)

$$K_{\text{quant}}^{2}(I)=(\frac{1}{12\mu (I{)}^{2}}),$$ (3b)

$$K_{\mathrm{cam}}^{2}(I)=(\frac{\sigma_{\mathrm{cam}}^{2}}{\mu (I{)}^{2}}).$$ (3c)

In Eq. (3a), γ is the analog to digital conversion ratio associated to the camera, which depends on the gain setting and the conversion factor (CF) of the camera, as $\gamma =\frac{\text{gain}}{\mathrm{CF}}$. In our investigations, the gain was set within a range of 1 to 72, corresponding to a 0 to 37 dB setting. The gain was tuned depending on the signal intensity. In 8-bit mode, the Basler camera we used had a conversion factor of CF=40.7. To reduce quantization noise, the gain of the camera was adjusted such that the camera readout grayscale values fell within the range of 40 to 255 at 8-bit recording unless the signal is too low. The camera noise $\sigma_{\mathrm{cam}}^{2}$ was estimated by calculating the variance of a series of 500 camera images recorded in the absence of any illumination sources. The blood flow index (BFI) can be related to $K_{\text{adjusted}}^{2}$ as^48^^,^^49^

$$\mathrm{BFI}=\frac{1}{K_{\text{adjusted}}^{2}}.$$ (4)

In all the results of this paper, we utilize the normalized BF index (normalized BFI) metric to provide normalized BF information for enhanced comparability across measurements (see Supplementary Material for more details about absolute BF measurement). Figure 2(c) shows a representative example of BF dynamics measured with our SCOS device located on the forehead. The BFI metric accounts for the total volume of blood moved in a given time period. According to classical fluid mechanics and Poiseuille’s law, BF can be expressed as $\mathrm{BF}=\frac{\delta P\pi r^{4}}{8\eta L}$, where δP represents the difference in blood pressure, r denotes the radius of the blood vessel, η is the dynamic viscosity of the blood, and L signifies the length of the blood vessel.^1^ Thus any alteration in the BFI means that there is a change in either the blood pressure or a change in the diameter of the blood vessel. It is worth noting that even a slight adjustment in the blood vessels’ radius can have a profound impact on BF due to the fourth power relationship with r. Such variations are especially significant as they accompany the modulation and regulation of BF.^2^

Note that the computational requirements needed to calculate the speckle contrast in Eqs. (1)–(4) from the recorded camera images can be handled by a standard consumer-grade computer [e.g., AMD 7950X CPU]. The most resource-demanding step is the normalization of the images and the calculation of $K_{\text{raw}}^{2}$ in Eq. (1), as the noise terms in Eq. (3) only need to be calculated once (precalibration) or were already calculated in Eq. (1). Therefore, the data recorded from our SCOS compact device can be processed and stored in real time using a dedicated Basler USB-PCIE card and SSD. It is also possible to expand the device to multiple channels.

## 3 Results and Discussion

**Fig. 3:**
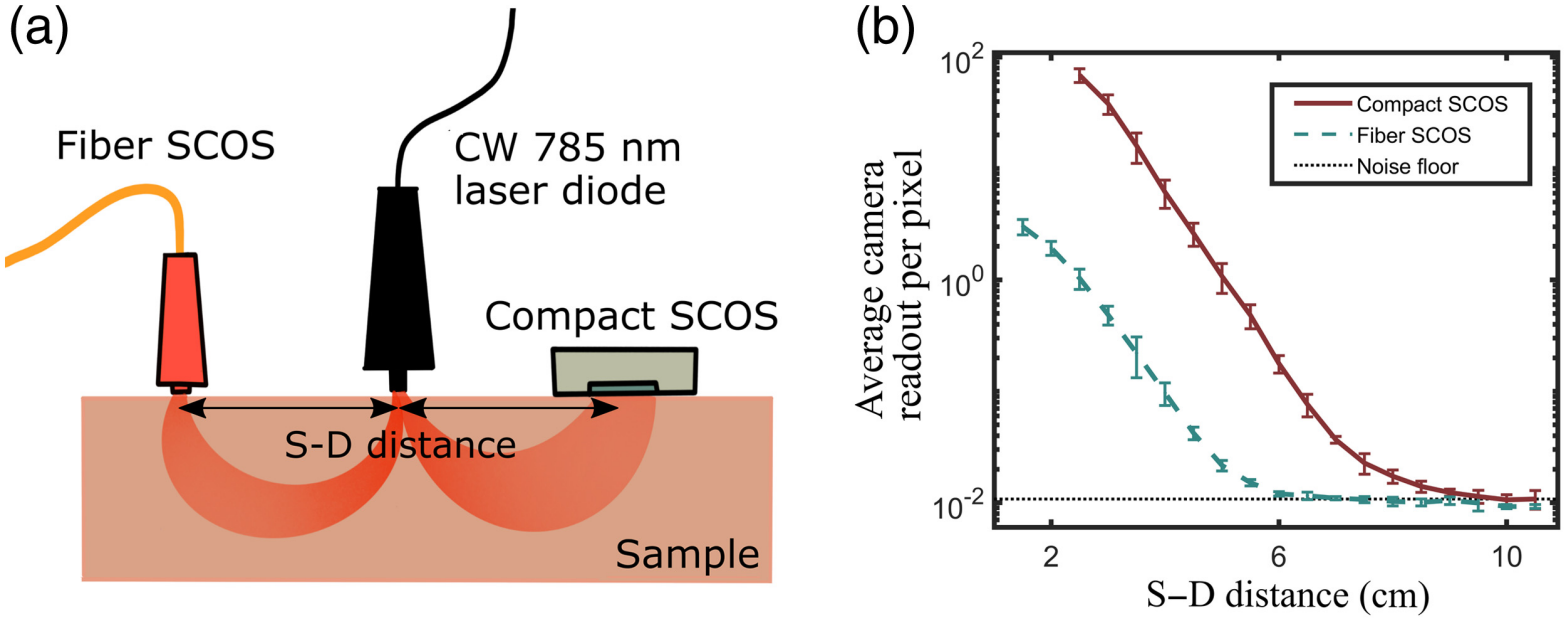
Experimental comparison between fiber SCOS and compact SCOS. (a) Overview of the experimental setup. (b) Average camera readout per pixel in analog-to-digital unit for fiber and compact SCOS measured at various S–D distances on a static sample. Notably, the compact SCOS demonstrates a significantly higher readout signal, averaging approximately 70 times more than its fiber counterpart. The fiber SCOS reaches the noise floor level at an S–D distance of ∼5.5  cm, whereas the compact SCOS maintains a robust signal up to S–D distance of 8.5 cm.

Relative to the single-fiber-based SCOS systems,^16^^–^^18^ the compact SCOS arrangement, where the sensor is directly positioned atop the region of interest, offers the larger collection area and NA of the sensor. This allows for two orders of magnitude increase in the number of photons collected. We compared the signal strength and stability of the compact SCOS and single-fiber-based SCOS systems. The experimental configuration is shown in Fig. 3(a) and features a continuous-wave 785-nm laser diode, acting as a common light source, and two SCOS detection modules symmetrically placed on each side of the laser source at the same S–D separation distance. On the one detection side, the compact SCOS was composed of a board camera [Basler daA1920-160um], directly positioned on the sample. On the opposing detection side, the fiber SCOS was composed of a 600-μm core diameter multimode optical fiber [Thorlabs FT600UMT], positioned on the sample. The other end of the fiber was coupled onto an identical camera to the one used in the compact version.^17^ We note that for our comparisons, we used a single fiber of 600-μm core diameter, but there exists systems with larger core multimode fiber (up to 1.5 mm) or with fiber bundles with improved signal collection.^26^^,^^33^

Theoretically, we expect the compact version to yield a signal gain of about 70 times compared to the fiber SCOS, as a result of the increased collection area. The camera sensor’s dimension is 6.6×4.1  mm, resulting in an approximate sensor area of $27\;{\mathrm{mm}}^{2}$ compared to the $0.28\text{-}{\mathrm{mm}}^{2}$ area of the 600-μm diameter multimode optical fiber, leading to about 95 times gain in detecting area. However, the camera sensor is positioned with a 5 mm to 7 mm gap from the sample, and the fiber is directly placed in contact with the sample. In this configuration, we calculated the NA of the camera as $\mathrm{NA}=n\;\sin(\theta )=n\frac{\frac{d}{2}}{\sqrt{(\frac{d}{2}{)}^{2}+z^{2}}}$, where n is the index of refraction of air [n=1.00], θ is the half-angle of the cone of light that enter the camera, d is the dimension (either in x or y) of the camera [$d_{x}=4.1\;\mathrm{mm}$ and $d_{y}=6.6\;\mathrm{mm}$], and z is the distance between the skin/sample and the camera [z=7  mm]. The NAs of the camera were calculated independently for the x and y directions since the sensor has different size in x and y. Based on the numbers, ${\mathrm{NA}}_{x}=0.28$ on one dimension and ${\mathrm{NA}}_{y}=0.42$ on the other dimension. The fiber has a numerical aperture of ${\mathrm{NA}}_{\text{fiber}}=0.39$. By taking into account the NA difference between the camera and fiber, we expect the collected signal gain between the compact SCOS over single-fiber SCOS to be $\frac{{\mathrm{NA}}_{x}\cdot {\mathrm{NA}}_{y}}{({\mathrm{NA}}_{\text{fiber}}{)}^{2}}\cdot \frac{{\text{Area}}_{\mathrm{CMOS}}}{{\text{Area}}_{\text{fiber}}}\approx 75$ times.

To experimentally validate this gain, we used a static sample (a block of meat) and measured the average camera readout per pixel at different S–D distances for the two detection systems. The camera readout per pixel was defined in analog-to-digital unit, which is the average grayscale readout of the camera per pixel. It can be converted to physical measurements such as flux or number of electrons, but since both cameras are the same model and the experiment is done with the same camera setting, directly comparing analog-to-digital unit is a fair comparison. The S–D distances ranged from 1.5 cm (2.5 cm) for the fiber (compact) SCOS to 10.5 cm. The size of the encasing blocks prevents using smaller S–D distances. The results are presented in Fig. 3(b) and were averaged over six different realizations at different locations. The error bars were estimated by calculating the standard deviation over the six different realizations. As shown, a consistent gain in the number of photons is captured by the compact SCOS over the fiber SCOS system across the multiple S–D distances. By calculating the signal ratio of the two, we determined that the compact version capture about 70 times more signal than its fiber-based SCOS counterpart. This significant improvement leads to an enhanced detectability at extended S–D distances, up to an S–D distance increase of 2.5 cm for the same signal readout in this case. Note that both devices reach the noise floor of the camera, although at different S–D distances. The fiber SCOS reaches the noise floor level at an S–D distance of ∼5.5  cm, whereas the compact SCOS maintains a robust signal even at a S–D distance of 8.0 cm on a static sample. These results showcase the superiority of compact SCOS over fiber SCOS in the ability to collect more signal, enabling the detectability of BF at extended S–D distances. Note that this increase has not yet considered the increase in stability by eliminating the potential fiber movement during the camera exposure time.

**Fig. 4:**
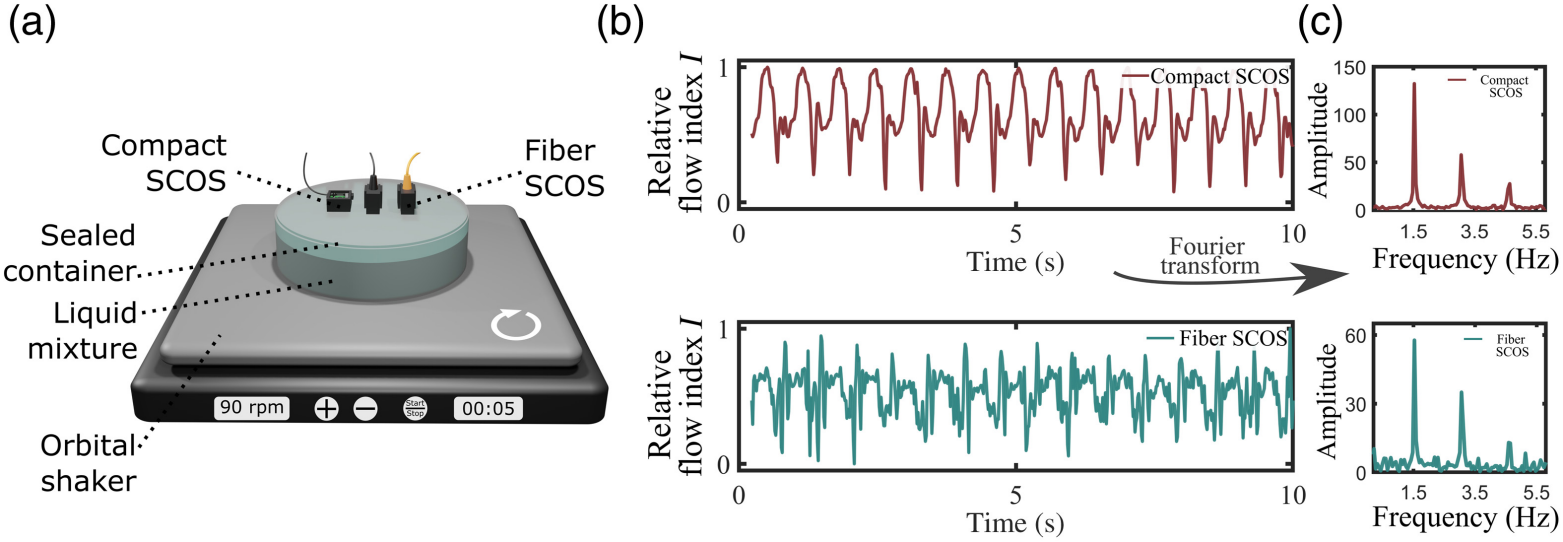
Experimental comparison of flow reproducibility between fiber SCOS and compact SCOS using a one-layer phantom rotating on an orbital shaker. (a) Experimental arrangement, (b) measured flow index, and (c) rotating frequencies obtained by Fourier transforming the flow signal in (b).

Next, we characterized the stability of the compact SCOS over the fiber SCOS. For that, we designed two distinct experiments where relative flow index values were calculated as in Eqs. (1)–(4) and Fig. 1. In the first experiment, presented in Fig. 4(a), both the compact and fiber SCOS systems were affixed on top of an one-layer phantom, which comprised of a sealed container filled with a liquid mixture (3D printing resin).^17^ The liquid mixture in the phantom was positioned on an orbital shaker (ONiLAB) set at a rotating speed of 90 rotations per min.^17^ The laser source, compact SCOS, and single-fiber SCOS systems were affixed to the phantom using the strap slots of the 3D-printed mounts (see Fig. 1 schematics). The phantom was affixed to the orbital shaker using straps, and the shaker having straps slots for affixing the phantom. A nonslip rubber mat was used for the best adhesion and stability. Both compact SCOS and fiber SCOS systems rotated synchronously with the liquid mixture. During each rotation, the SCOS systems are measuring the change of decorrelation time within the liquid mixture.^17^ In this context, the SCOS systems measure a liquid flow dynamic similar to the blood flow dynamic for humans, see Appendix A of Ref. 17. The compact SCOS and fiber SCOS were set at S–D distances that yielded an equivalent photon count. In this configuration, the light power collected by the compact SCOS and fiber SCOS detection systems are equivalent. Consequently, we anticipate assessing the stability of the compact SCOS and fiber SCOS by comparing the recorded liquid flow from the rotating phantom. The results are shown in Fig. 4(b). As expected, the liquid flow measured by the compact SCOS exhibits superior signal quality compared to that obtained by the fiber SCOS. This observation is further validated when examining the frequencies present in the Fourier spectrum of the flow index signal I, [Fig. 4(c)]. The Fourier amplitude peak, centered around 1.5 Hz, corresponds to the rotational frequency of the orbital shaker (90 rotations per minute, translating to 1.5 rotations per second). However, the noise level is slightly higher in the fiber SCOS Fourier spectrum, indicating that the measured flow index from the fiber SCOS is less reproducible than that from compact SCOS. To quantitatively evaluate the reproducibility of the measured liquid flow signal, we computed the Pearson correlation factor for each SCOS system as

$$\rho (I(t),I(t+dt))=\frac{\sum_{t=1}^{T}(I(t)-\overset{¯}{I})(I(t+dt)-\overset{¯}{I})}{\sqrt{\sum_{t=1}^{T}(I(t)-\overset{¯}{I}{)}^{2}\sum_{t=1}^{T}(I(t+dt)-\overset{¯}{I}{)}^{2}}},$$ (5)

where I(t) is the measured flow signal in Fig. 4(a), $\overset{¯}{I}$ is the mean flow index, and I(t+dt) is the signal shifted by one period of dt=1/1.5  Hz=0.66  s. The compact SCOS correlation factor was $\rho_{\text{compact}}=0.94$ and the fiber SCOS was $\rho_{\text{fiber}}=0.67$, demonstrating that the measured periodic signal from the compact SCOS is significantly more stable than its fiber counterpart.

**Fig. 5:**
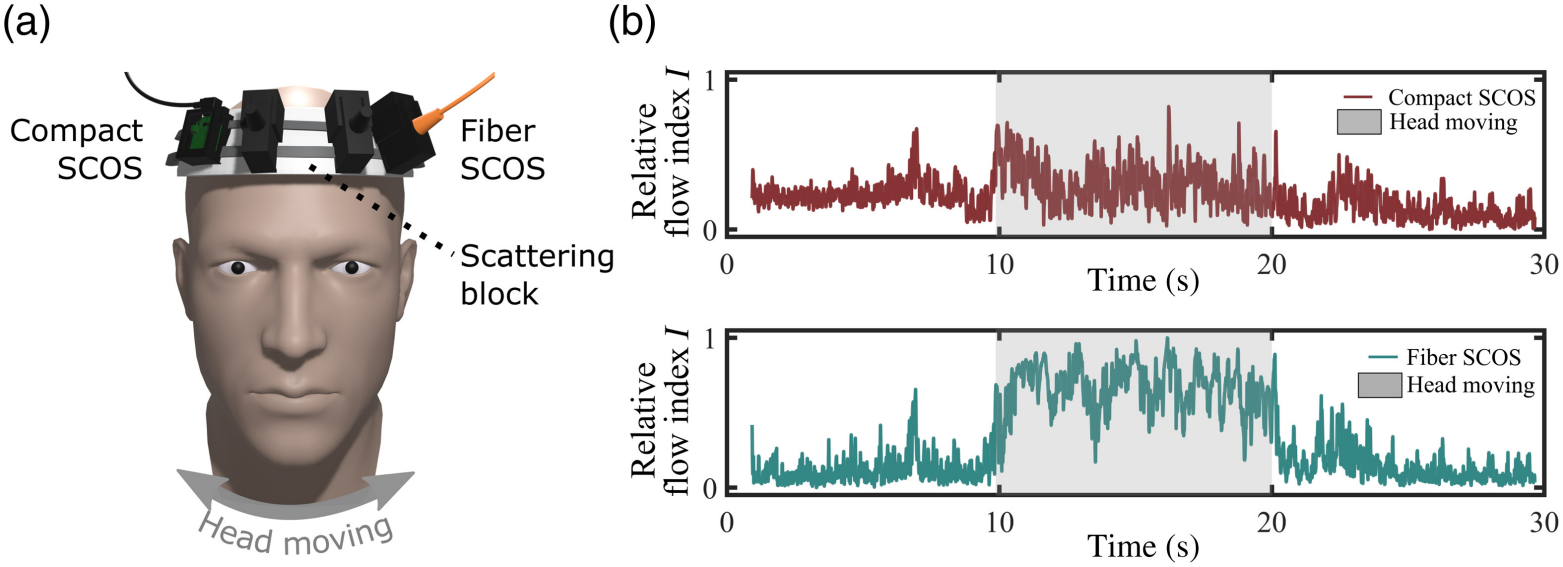
Experimental comparison of stability between fiber SCOS and compact SCOS using a static scattering block on the forehead of a subject. (a) Experimental arrangement and (b) measured flow index where the head is moving from 10 to 20 s.

Next, we further investigated the robustness of the systems against human head movements, presented in Fig. 5(a). To conduct this assessment, both SCOS systems were positioned on the forehead of a subject, with a static scattering block interposed between the SCOS setups and the subject’s forehead. The static scattering block comprised a rigid block of packaging foam, complemented by a thick layer of black tape on its backside to prevent any laser light from entering the subject’s head. Consequently, the SCOS systems exclusively detected light interacting with the static scattering block. The S–D distances of both the compact SCOS and fiber SCOS are equivalent, in order to replicate CBF data acquisition scenarios. As a result, the signal intensity on the fiber SCOS is about 70 times lower than that of the compact SCOS. The measurement was performed over a 30 s interval, following a specific protocol: from 0 to 10 s, the subject maintained a still position; at the 10 s mark, the subject was asked to laterally move their head (left to right and right to left) for the subsequent 10 s; and finally, the subject resumed a stationary position for the remaining 10 s. The results are presented in Fig. 5(b) and show that the flow measured by the compact SCOS exhibits less noise movement that with the fiber SCOS during head movements. In addition, the overall flow index notably rises due to the SCOS systems’ movements accompanying head motions. As shown in Fig. 5(b), this increase in flow is more pronounced for the fiber SCOS than the compact SCOS, indicating than the compact SCOS experiences less movement vibrations than the fiber SCOS during head motions. The more prominent shift in the fiber SCOS is due to additional decorrelation resulting from fiber movement leading to lower contrast and higher flow. Without the fiber, this source of instability is largely eliminated, resulting in the compact SCOS displaying more stable flow measurements during movements.

The results shown in Figs. 4 and 5 demonstrate that despite both systems being affected by noise induced by head motion, compact SCOS exhibits superior stability compared to fiber SCOS. In fiber SCOS, two primary factors contribute to noise during head motion: the subtle movements of the fiber tip in contact with the sample and the amplified mode mixing of laser light within the vibrating fiber. Compact SCOS is more resilient to sensor movement in contact with the sample due to its larger surface contact and larger sensor size. Compact SCOS does not suffer from mode mixing. Additionally, there are other sources of noise during head movement common to both systems, such as the laser source movement will alter the trajectories of the laser light in the sample and result in transmitted speckle pattern movements.

To determine the capability of the compact SCOS system to detect blood flow at large S–D distances, we further tested the two SCOS systems by measuring blood flow on the forehead of a human subject at S–D distances ranging from 3 to 5.5 cm. In order to assess the CBF dynamics, we need to adjust the system so that the system detects a larger portion of signal from the brain than the scalp + skull layers. It was shown that laser light propagating inside the human head follows a “banana” spatial sensitivity profile, which extend deeper into the head as the S–D distance increases.^12^^–^^14^^,^^17^^,^^35^^,^^36^ The brain sensitivity was reported to significantly increase with the depth of penetration, i.e., by increasing the S–D >3  cm. Note that, even at such large S–D distance, the collected signal would still be influenced by blood flow in the scalp. Some techniques such time-gating^50^ or calibration via short S–D measurement^51^ were previously proposed to filter out the signal from the scalp and skull layers.

**Fig. 6:**
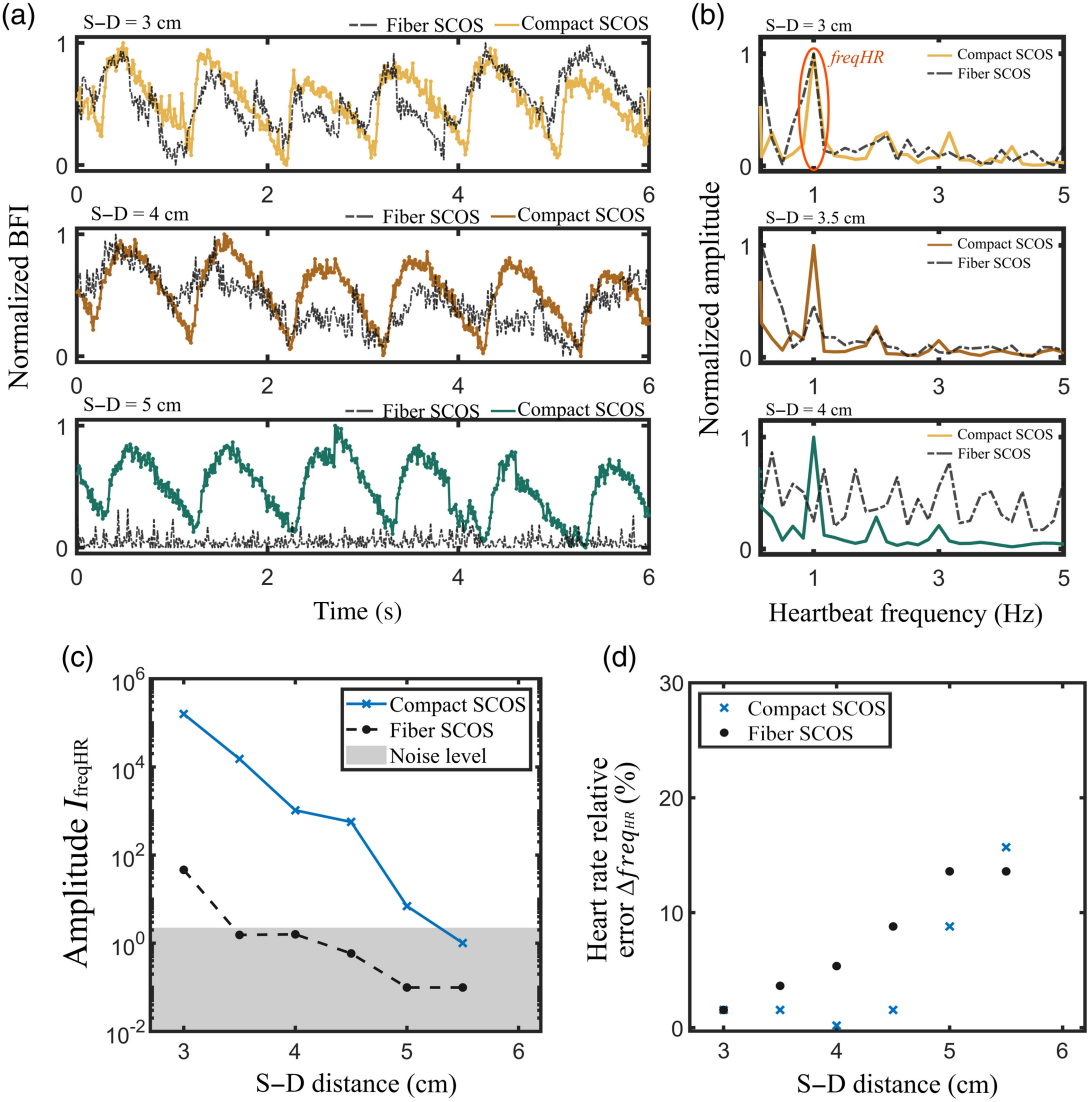
Experimental comparison between fiber SCOS and compact SCOS in blood flow measurement. (a) Measured blood flow at different S–D distances on the forehead of a subject. (b) Heart rate frequencies obtained by Fourier transforming the blood flow signal in (a). (c) Amplitude of the heartbeat frequency peak $I_{\mathrm{freqHR}}$ as a function of the S–D distance. (d) Heart rate relative percentage error between SCOS and with a pulse oximeter. In (c) and (d), the results were averaged over three realizations.

The results are shown in Fig. 6. Figure 6(a) shows the measured blood flow by the compact and fiber SCOS system at S–D distances of 3, 4, and 5 cm. Figure 6(b) shows the normalized Fourier transform of the blood flow signal in Fig. 6(a). The Fourier amplitude peak centered around HR = 1 Hz corresponds to the heart rate amplitude peak of the subject.^17^ We verified that the heart rate of the subject matches with the one measured from a standard pulse oximeter.

The quality of the measured blood flow signal can be assessed by examining the amplitude of the heart rate Fourier peak.^17^ Figure 6(c) shows the amplitude of the heart rate Fourier peak at different S–D distances ranging from 3 to 5.5 cm for both the compact and fiber SCOS. The results were averaged over three realizations. As shown, the compact SCOS exhibits a significant gain over the fiber SCOS system across the S–D distances. Finally, the heartbeat of the subject can be measured by measuring the frequency of the heart rate ${\mathrm{freq}}_{\mathrm{HR}}$ Fourier peak.^17^ The measured SCOS heart rate ${\mathrm{freq}}_{\mathrm{HR}}^{\text{SCOS}}$ can be compared with the one measured from a standard pulse oximeter ${\mathrm{freq}}_{\mathrm{HR}}^{\text{oxymeter}}$ by calculating the relative percentage error as

$$\Delta {\mathrm{freq}}_{\mathrm{HR}}=\frac{\left|{\mathrm{freq}}_{\mathrm{HR}}^{\text{oxymeter}}-{\mathrm{freq}}_{\mathrm{HR}}^{\text{SCOS}}\right|}{{\mathrm{freq}}_{\mathrm{HR}}^{\text{oxymeter}}}\cdot 100\%.$$ (6)

**Fig. 7:**
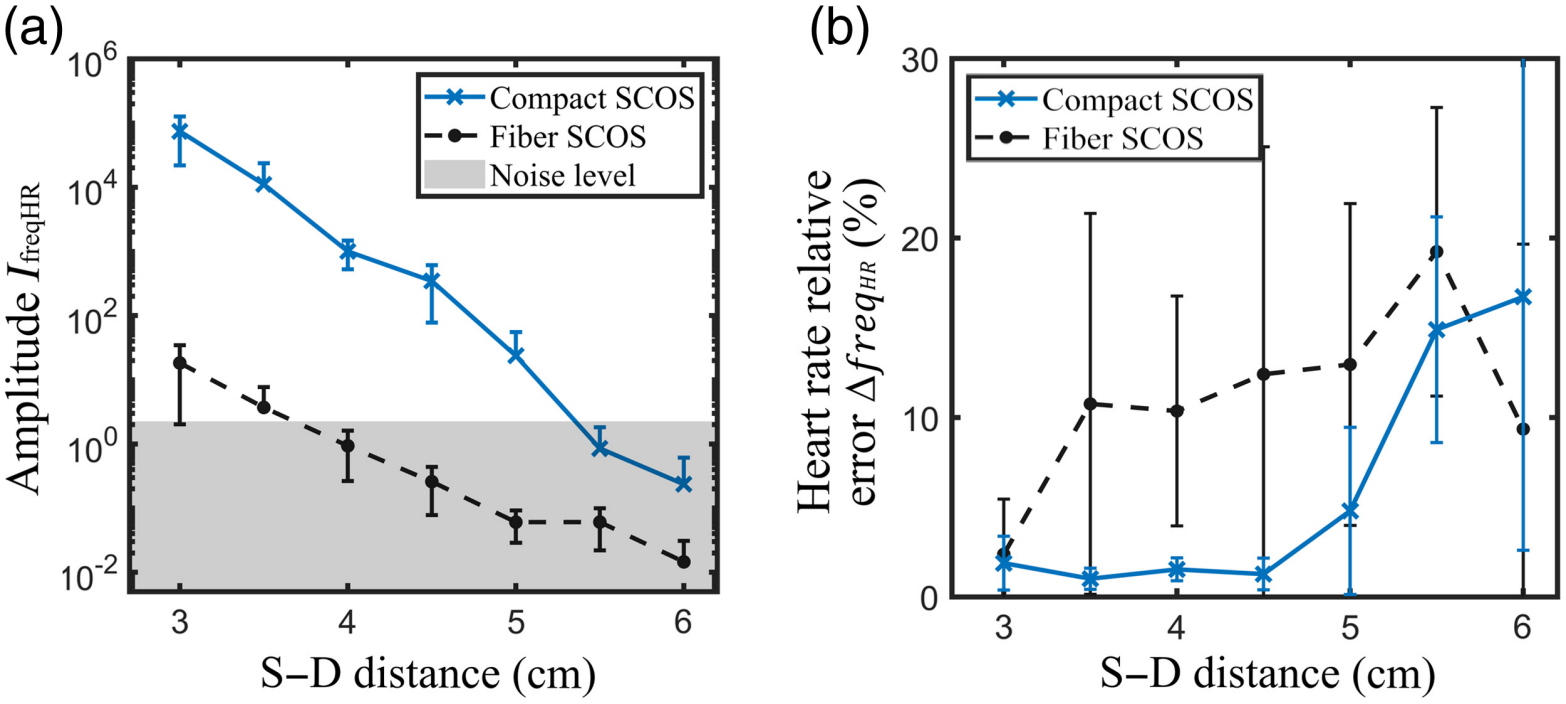
Experimental comparison between fiber and compact SCOS in blood flow measurement on a cohort of five subjects. (a) Averaged amplitude of the heartbeat frequency peak and (b) heart rate relative error as a function of the S–D distance.

Figure 6(d) shows the heart rate relative percentage error across the S–D distances for both the compact and fiber SCOS systems. As shown, the compact SCOS exhibits a lower error than the fiber SCOS system within S–D distances from 3 to 5 cm. Finally, the measurements of Fig. 6 were repeated on a cohort of five subjects. The results are shown in Fig. 7, by showing the amplitude of the heart rate Fourier peak and the heart rate relative error at different S–D distances for both the compact and fiber SCOS. For each subject, three realizations were recorded. The results were averaged over the five different subjects, and the error bar was determined by calculating the standard deviation. Across the five subjects, compact SCOS shows a significant gain in signal over the fiber SCOS, measuring CBF up to a S–D distance of 5 cm. Based on Fig. 7(a), the amplitude $I_{\mathrm{freqHR}}$ for compact SCOS at S–D distance of 5 cm is comparable to a S–D of 3 cm for fiber SCOS. This is further validated by the correct prediction of the heart rate frequency at large S–D distances, as shown in Fig. 7(b). The pronounced error bars observed at larger S–D distances result from the signal being heavily masked by noise, leading to inconsistent and unreliable heart rate predictions across different realizations and subjects. The typical measured blood flow signals across the five subjects are shown in the Supplementary Material.

## 4 Conclusion

We have characterized a compact and cost-effective laser-powered device for assessing CBF up to a S–D distance of 5 cm. The device is based on SCOS technology, which is derived from LSCI. The hardware consists of only two components: a laser diode and a CMOS-based board camera. It offers real-time blood flow monitoring at an 80 Hz sampling rate while maintaining a lightweight and modular design with an ∼70-fold improvement in collected signal over single-fiber-based SCOS. We demonstrated that the device could measure CBF up to a source-to-detector distance of 5 cm across a cohort of five subjects. We aim to apply this new device to the estimation of cerebrovascular reactivity, by measuring the ability of the brain to adjust CBF in response to oxygen supply changes within the body, including the amplitude of blood flow modulation and the speed at which blood flow returns to baseline. Ultimately, this device has potential applications in both clinical assessment of cerebrovascular diseases and in basic research of the hemodynamic response to brain activity.

## Supplementary Material



## Data Availability

The data that support the findings of this study are available from the corresponding author upon reasonable request.
