# Supplementary material for: Compact and cost-effective laser-powered speckle contrast optical spectroscopy fiber-free device for measuring cerebral blood flow
Source: J Biomed Opt. 2024 May 31;29(6):067001. doi: 10.1117/1.JBO.29.6.067001 (PMC11140771; doi:10.1117/1.JBO.29.6.067001)
Supplement: Supplementary file 1 [file JBO_029_067001_SD001.pdf]

Supplementary Material:

A compact and cost-effective laser-powered speckle contrast optical spectroscopy fiber-free device for measuring cerebral blood flow.

Yu Xi Huang,[a,†] Simon Mahler,[a,†] Maya Dickson,[a] Aidin Abedi,[b] J. Michael Tyszka,[c] Yu Tung Lo,[b] Jonathan Russin,[b,d] Charles Liu,[b,d,*] Changhuei Yang[a,**]
[a] Department of Electrical Engineering, California Institute of Technology, Pasadena, CA 91125, USA
[b]USC Neurorestoration Center and Department of Neurological Surgery, University of Southern California, Los Angeles, CA 90033, USA
[c]Division of Humanities and Social Sciences, California Institute of Technology, Pasadena, CA 91125, USA
[d]Rancho Los Amigos National Rehabilitation Center, Downey, CA 90242, USA
[†]These authors contributed equally to this work.
*Email: cliu@usc.edu **Email: chyang@caltech.edu

Pre-processing and normalization of the images

One key step in Fig. 2 is to remove the non-uniform intensity distribution (inhomogeneities) of the camera images before speckle contrast calculations. In Fig. S1, we show the method and corresponding result.

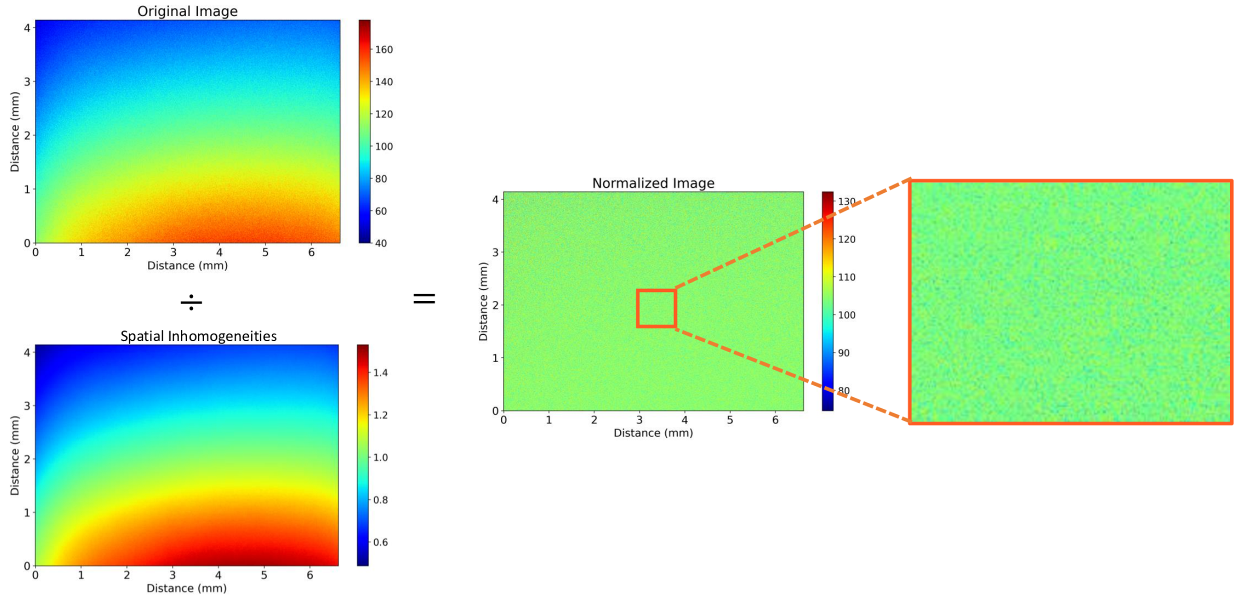

Fig. S1 Removing the inhomogeneities from the measured patterns (normalization) before speckle contrast calculations.

As shown in Fig. S1, the recorded raw image exhibits a pronounced intensity gradient, due to the difference (about 4 mm) in S-D distances between the two edges of the camera sensor from the source. To calibrate this, we captured 300 images before each measurement, from which a mean image was computed. To obtain the image inhomogeneity, the mean image is then normalized such that its average is set to unity. Before calculating the speckle contrast, each raw image is divided by the image inhomogeneity, removing the intensity gradient.

Discussion on speckle size and SNR

In the main text, we estimated the number of speckles per pixel to be about 10 speckles per pixels, corresponding to a one-dimensional speckle-to-pixel length ratio s/p = 0.3. It is true that to

accurately image speckles, one should adhere to the Nyquist sampling theorem of having a speckle-to-pixel ratio (s/p) of 2, meaning that the speckle size is twice the pixel pitch of the camera. However, for extracting the dynamics of the scattering medium via speckle contrast calculations, this strict requirement of s/p ≥ 2 is relaxed, as demonstrated in [1-3]. The SNR was defined as the ratio between the measured squared speckle contrast K^2 over all the noise contributions to the squared speckle contrast $\sigma(K^2)$ [2]:

$$SNR = \frac{K^2}{\sigma(K^2)}.$$

In our experimental setup, the s/p ratio was approximately 0.3 (i.e. 10 speckles per pixel), which is an optimal s/p ratio for achieving high SNR, see Fig. 2. To calculate our s/p ratio, we assumed the free space propagation of speckles at a distance z from the sample and an aperture size of d. With this assumption, the speckle length is $s = \frac{\lambda z}{d}$. In our case, $d = 5.3$ mm is the average dimension (in x and y) of the camera sensor [d_x= 4.1 mm and d_x= 6.6 mm], z = 7 mm is the distance between the skin/sample and the camera and λ = 785 nm is the illumination wavelength. Those numbers lead to a speckle size of $s = 1.04$ mm. The pixel pitch of our camera (Basler daA1920-160um) was $p = 3.45$ µm, leading to a s/p ratio of 0.3.

To further prove our point, we tuned the model presented in [2] with parameters according to our experimental setup (i.e. with camera Basler daA1920-160um). We simulated two averaged photon flux per speckle levels of 1,000, 10,000, and 100,000 photons/speckle/sec. The results, presented in Fig. S2, shows that the SNR is optimal at s/p between 0.2 and 0.7.

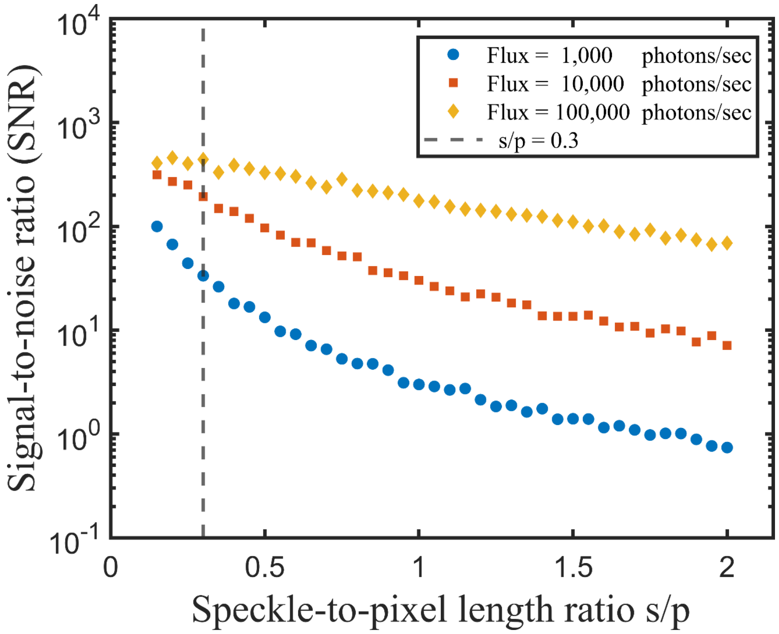

Fig. S2 Simulation results showing relationship between s/p ratio and SNR at different photon flux levels.

At lower signal regime (< 10,000 photons/sec), which is typically the situation at large S-D distances, smaller s/p ratio will yield a higher SNR. This is intuitive as higher s/p ratio would limit the total number of photons captured by the sensor. In signal-starved regime, more photons are essential to combat the camera noise and other sources of instability.

CBF measurement results on five subjects

Figure S1 shows typical normalized BFI measurement results of the compact speckle contrast optical spectroscopy (SCOS) system at different source to detector (S-D) distance on the five subjects of Fig. 7.

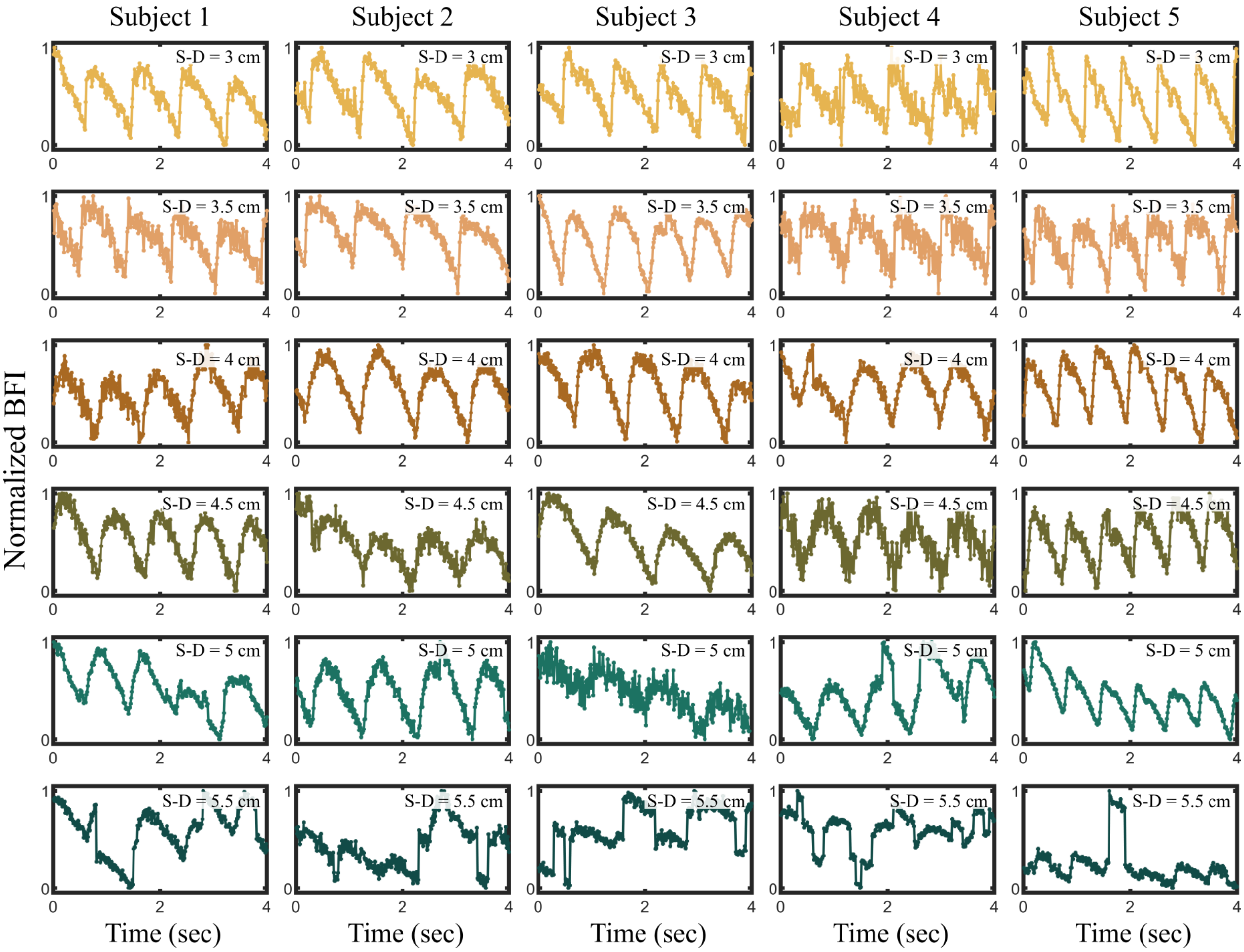

Fig. S3 Blood flow measurement results on the forehead of the five subjects in Fig. 7 at different S-D distances.

Study participation

We tested and experimented our device across a cohort of five subjects. Participants for this study were recruited from the Caltech and Pasadena community, selected among adult human aged from 21 to 65 years. Prior to the experiments, each participant completed a health questionnaire, and their blood pressure was recorded. The human research protocol for this study received approval from the Caltech Institutional Review Board (IRB). The total illumination power was limited to 45 mW to ensure that the laser light intensity level of the area of illumination is well within the American National Standards Institute (ANSI) laser safety standards for maximum permissible exposure (2.95 mW/mm2) for skin exposure to a 785nm laser beam.

To simplify the experiment and implementation of the device, SCOS was conducted on hairless areas, such as the forehead or frontotemporal region. Optical transmission is optimal when both the light source and detector are positioned on hairless regions of the head. An ideal scenario entails a hair-free circular space of 0.5 cm diameter for the illumination spot and a square area of 1 cm × 1 cm for the detection device. While these requirements are manageable, they may pose inconveniences for participants with hair who are unwilling to shave. To mitigate this, as further step, we aim to design and use of 3D-printed mounts equipped with hair separators. This innovative solution aims to minimize hair interference with the optical transmission process, enhancing the device's usability and accommodating participants with hair.

Absolute decorrelation time measurement

After obtaining $K^2_{adjusted}$, as in Eq. (2) in the manuscript, one can extract the decorrelation time τ as[1,2]:

$$K^2_{adjusted} = \frac{\beta\tau}{T}\left[1 + \frac{\tau}{2T}\left(\exp\left(-\frac{2T}{\tau}\right) - 1\right)\right], \qquad (S1)$$

where T is the exposure time of the camera, and β is a constant that accounts for the loss of correlation associated with the ratio of the detector size to the speckle size and polarization. At low signal, β may deviate from typical calibration due to high sensitivity to noise. The correlation factor β was determined by measuring the speckle contrast with our compact SCOS device on a static sample (here a block of meat). The β value was $\beta \approx 0.05$, which is relatively low because the average speckle size is smaller than the pixel size, resulting in multiple speckles per pixel. Note that for relative cerebral blood flow measurements or normalized blood flow index, β can be disregarded.

In SCOS, the detecting device operates with an exposure time significantly greater than the decorrelation time of the sample, i.e. $T \gg \tau$. In our case, $T = 6$ ms. Consequently, Eq. $(S1)$ simplifies to:

$$K^2_{adjusted} \approx \frac{(\beta)\tau}{T}. \qquad (S2)$$

Then, the blood flow index (BFI) can also be related to $K^2_{adjusted}$ and τ as[3,4]:

$$BFI = \frac{1}{K^2_{adjusted}} \approx \frac{T}{(\beta)\tau}. \qquad (S3)$$

References

1. J. Xu, A. K. Jahromi, and C. Yang, "Diffusing wave spectroscopy: A unified treatment on temporal sampling and speckle ensemble methods," APL Photonics **6**(1), 016105 (2021) [doi:10.1063/5.0034576].
2. S. Zilpelwar et al., "Model of dynamic speckle evolution for evaluating laser speckle contrast measurements of tissue dynamics," Biomed. Opt. Express **13**(12), 6533 (2022) [doi:10.1364/BOE.472263].
3. M. B. Robinson et al., "Comparing the performance potential of speckle contrast optical spectroscopy and diffuse correlation spectroscopy for cerebral blood flow monitoring using Monte Carlo simulations in realistic head geometries," Neurophoton. **11**(01) (2024) [doi:10.1117/1.NPh.11.1.015004].
